
JOURNAL INFORMATION
==============================
NLM Title Abbreviation: ScientificWorldJournal
Journal ID: ScientificWorldJournal
NLM Journal ID: 101131163
Journal ID: TSWJ

The Scientific World Journal

ISSN: 2356-6140
EISSN: 1537-744X
Publisher: Wiley

ARTICLE INFORMATION
==============================
PMCID: PMC5848853
PMID: 18956116
DOI: 10.1100/tsw.2008.119
Article ID: 745274
Article version: 1
Subjects: Editorial

Implementation of a Positive Youth Development Program in Hong Kong using Seven Cases as Evidence

Shek Daniel T. L. 1 2 3 *
Ma Hing Keung 4
1 Quality of Life Centre, Hong Kong Institute of Asia-Pacific Studies, The Chinese University of Hong Kong, Hong Kong
2 Kiang Wu Nursing College of Macau, Macau
3 Social Welfare Practice and Research Centre, The Chinese University of Hong Kong, Hong Kong
4 Department of Education Studies, Hong Kong Baptist University, Hong Kong
*Daniel T. L. Shek: danielshek@cuhk.edu.hkAcademic Editor: Joav Merrick

Publication date: 2008
Electronic publication date: 2008 Oct 10
Volume: 8
First page: 976
Last page: 979
Received 2008 Jun 18; Revised 2008 Aug 18; Accepted 2008 Aug 23
Copyright: Copyright © 2008 Daniel T. L. Shek and Hing Keung Ma.
Copyright year: 2008
License: This is an open access article distributed under the Creative Commons Attribution License, which permits unrestricted use, distribution, and reproduction in any medium, provided the original work is properly cited.
License URL: https://creativecommons.org/licenses/by/3.0/

Funding: The Hong Kong Jockey Club Charities Trust

==============================
Editorial
Special Issue: Implementing Positive Youth Development Programs: Case Studies from Project P.A.T.H.S. in Hong Kong
*TheScientificWorldJOURNAL* (2008) 8, 976–979
TSW Holistic Health & Medicine
ISSN 1537-744X; DOI 10.1100/tsw.2008.119

TheScientificWorldJOURNAL
www.thescientificworld.com

### Implementation of a Positive Youth Development Program in Hong Kong using Seven Cases as Evidence

Daniel T.L. Shek[1,2,3,*] and Hing Keung Ma[4]

*[1]Quality of Life Centre, Hong Kong Institute of Asia-Pacific Studies, The Chinese University of Hong Kong; [2]Kiang Wu Nursing College of Macau; [3]Social Welfare Practice and Research Centre, The Chinese University of Hong Kong; [4]Department of Education Studies, Hong Kong Baptist University*

**E-mail**: danielshek@cuhk.edu.hk

*Received June 18, 2008; Revised August 18, 2008; Accepted August 23, 2008; Published October 10, 2008*

---

In Western contexts, curricular-based positive youth development programs have been designed to promote and nurture the potentials and competencies in young people. Typically, several steps are commonly involved in the development of positive youth development programs. According to Weissberg et al.[1], there are five stages that are intrinsic to the overall framework underlying the conceptualization, design, implementation, evaluation, and dissemination of school-based social competence promotion programs. The first stage, "conceptualization", involves the identification of personal resources (such as skills, knowledge, and beliefs) and environmental support, and their combinations that promote social competence in specified domains and/or prevent mental health problems based on theories, research findings, and intervention experiences. The second stage is "design", in which developmentally and culturally appropriate programs with application of effective classroom teaching principles are developed. In such programs, replicable curricula that clearly describe the following areas are developed: (1) how students are taught the targeted personal resources and (2) how real-life opportunities are structured in order to practice and reinforce those taught skills. The third stage is "implementation", where the program is adapted to the ecology of the school setting and where mechanisms are used to monitor the program integrity. The fourth stage is "evaluation", where the researchers examine whether (1) the program is implemented effectively, (2) the program improves the personal resources of the students, and (3) there are different combinations of personal and environmental factors that are related to program effectiveness. The fifth stage is "maintenance and dissemination", where issues of sustainability of the program and dissemination are considered.

Similarly, Battistich et al.[2] proposed that there were five tasks required for effective and enduring implementation of primary prevention programs. These tasks include program conceptualization, program design, program implementation, program monitoring and evaluation, and program diffusion. Weissberg and Elias[3] also suggested four stages of program development, including conceptualization, design, implementation, and institutionalization.

Among these components in positive youth development programs, the implementation stage can be considered as a crucial component. Obviously, programs with elegant and evidence-based conceptualization and program design will be futile if they are not implemented in a proper manner. Practically, there may be policies and personnel factors that inhibit the healthy implementation of the program, thus creating a Type III error (i.e., existence or nonexistence of program effect because of the occurrence of activities different from those intended by the program developers).

*Corresponding author.
©2008 with author.
Published by TheScientificWorld; www.thescientificworld.com

To promote holistic development among adolescents in Hong Kong, The Hong Kong Jockey Club Charities Trust approved HK$400 million to launch a project entitled "P.A.T.H.S. to Adulthood: A Jockey Club Youth Enhancement Scheme". The word "P.A.T.H.S." denotes **P**ositive **A**dolescent **T**raining through **H**olistic **S**ocial Programmes. There are two tiers of programs in this project (Tier 1 and Tier 2). The Tier 1 Program is a curricula-based positive youth development program where students in Secondary 1 to 3 participate in the program, normally with 20 h of training in the school year in each grade[4]. The Tier 2 Program targets adolescents with greater psychosocial needs.

To understand the implementation details of the Tier 1 Program, a series of case studies were carried out based on the involvement of seven schools. Baker[5] pointed out that "a case study is a research strategy which focuses on single organization, institution, event, decision, policy, or group (or possibly a multiple set) which is not a specific method of social research" (p. 299). Yegidis and Weinbach[6] commented that although case studies are often misunderstood as "less scientific", they are particularly useful when (1) little is known about the area being studied, (2) topics involving illegal and/or socially sanctioned behavior are studied, and (3) it is impossible to draw a representative sample of participants. Despite the problem of low generalizability, case studies can help to achieve insight and new knowledge.

To look at the quality of implementation from different angles, seven cases were employed in this study. In the first case reported by Shek and Sun, the 20-h Tier 1 Program was covered in the class teachers' periods, the Life Education subject, and project-based learning. The findings showed that several factors related to policy, people, program, process, and place (5 "P"s) facilitated the implementation process of the Tier 1 Program in the school. Based on the same model, obstacles and difficulties that affected the quality of implementation were also identified. Furthermore, implications of the findings on future program implementation are highlighted.

In contrast to the first case, the second case reported by Shek, Chak, and Chan was based on a school in which the Tier 1 Program (20 h) was wholly incorporated into the Life Education subject of the formal curriculum. Results showed that several school-related factors facilitated the implementation of the Tier 1 Program in the school, including administrative support from the school and social work agency, good selection of dedicated teachers, positive perception of the program among teachers, good use of the teachers' self-disclosure, effective continuous assessment, and excellent coteaching relationships. Difficulties encountered by the school in the process of implementation were also identified.

In the third case, Lee reported a case study based on a school in which the 20-h Tier 1 Program was integrated and delivered in Liberal Studies lessons; the teacher implemented the program with the assistance of a teaching assistant. With reference to the changing educational policy environment, the school carried out school policy and structural changes to facilitate the implementation of a new curriculum that was unfamiliar to the teachers. Generally speaking, the quality of program implementation in the school was good and the program was well received by the program implementers.

The fourth case conducted by Lam was based on a school that admitted students with high academic achievement. Findings in this case are particularly important because there is a common myth that students with high academic achievement do not need curricula-based positive youth development programs. Similar to other cases, the results suggested that clear vision and program goals, high quality of curriculum, helpful leadership, positive teacher attitude, and strong administrative support were conducive to program success. In addition, the program was perceived in a positive light by the program implementers and participants, and the program was seen as beneficial to the program participants.

In contrast, in the fifth case, also conducted by Lam, data were collected from a school that admitted students with low academic achievement. Findings in this case are particularly important because there is also a common myth that curricula-based positive youth development programs do not work with students with low academic achievement who usually display behavioral and emotional problems. The Tier 1 Program was incorporated into the formal curriculum, such as Citizen and Moral Education lessons. Results showed that administrative and "people" factors facilitated success in the implementation process, such as commitment of the program implementers, positive teacher beliefs, leadership, positive school culture, prior planning, policy and administrative support, and program ownership.

In the sixth case study, Lee reported how the experience gained in the Experimental Implementation Phase (where the Tier 1 Program was implemented in around 50 schools[7]) could be used to improve program implementation quality in the Full Implementation Phase (where the Tier 1 Program was implemented in more than 200 schools[4]). Several factors that facilitated the implementation of the program were identified, including adoption of an incremental change strategy, inclusion of the program into both formal and informal curricula, positive perceptions of the program among staff and agency social workers, adequate school administrative support, excellent cooperation between the school and social work agency, presence of a dedicated school contact person, instructors who engaged themselves in continuous quality improvement of the implementation, and emphasis on application of what has been learned.

In the final case by Sun, Shek, and Siu, interviews with the school principal, vice-principal, and social worker were conducted in order to understand the macroschool and microclassroom environmental factors conducive to success in program implementation. Results showed that several positive school and classroom attributes were conducive to program success, including positive school culture and belief in the students' potential, inviting school environment, encouraging classroom environment, high involvement of school administrative personnel, and systematic program arrangement.

To understand the factors that facilitate or impede the implementation quality of the Tier 1 Program of the Project P.A.T.H.S., cross-case analyses were carried out. Shek and Sun identified several factors related to policy, people, program, process, and place that influenced the quality of program implementation, particularly those related to policy and people. Furthermore, the findings showed that incorporation of the program into the formal curriculum was a viable arrangement and the program could benefit students with different academic achievement levels.

In view of the paucity of research examining the factors that influence the implementation of positive youth development programs, particularly in the Chinese culture[7], the present case studies are pioneering additions to the literature. Generally speaking, different factors in respect to policy, people, program, process, and place influence the implementation quality of the programs concerned. These findings can obviously help to develop conceptual models on the determinants of quality of implementation of positive youth development programs in the global and Chinese contexts. These findings also have practical implications on how the implementation quality of positive youth development programs can be improved.

### ACKNOWLEDGMENTS

The preparation for this paper and Project P.A.T.H.S. were financially supported by The Hong Kong Jockey Club Charities Trust.

### REFERENCES

1. Weissberg, R.P., Caplan, M.Z., and Sivo, P.J. (1989) A new conceptual framework for establishing school-based social competence promotion programs. In *Primary Prevention and Promotion in Schools*. Bond, L.A. and Compas, B.E., Eds. Sage, Newbury Park, CA. pp. 255–296.
2. Battistich, V.A., Elias, M.J., and Branden-Muller, L.R. (1992) Two school-based approaches to promoting children's social competence. In *Improving Children's Lives: Global Perspectives on Prevention*. Albee, G.W., Bond, L.A., and Monsey, T.V.C., Eds. Sage, Newbury Park, CA. pp. 217–234.
3. Weissberg, R.P. and Elias, M.J. (1993) Enhancing young people's social competence and health behavior: an important challenge for educators, scientists, policymakers, and funders. *Appl. Prev. Psychol.* **2,** 179–190.
4. Shek, D.T.L. and Ma, H.K. (2007) Subjective outcome evaluation of the Project P.A.T.H.S.: findings based on the perspective of the program participants. *TheScientificWorldJOURNAL* **7,** 47–55.
5. Baker, T.L. (1994) *Doing Social Research*. McGraw-Hill, New York.

6. Yegidis, B.L. and Weinbach, R.W. (2006) *Research Methods for Social Workers*. Pearson, Boston.
7. Shek, D.T.L., Ma, H.K., Lui, J.H.Y., and Lung, D.W.M. (2006) Process evaluation of the Tier 1 Program of the Project P.A.T.H.S. *TheScientificWorldJOURNAL* **6,** 2264–2273.

**This article should be cited as follows:**

Shek, D.T.L. and Ma, H.K. (2008) Implementation of a positive youth development program in Hong Kong using seven cases as evidence. *TheScientificWorldJOURNAL:* TSW Holistic Health & Medicine **8,** 976–979. DOI 10.1100/tsw.2008.119.
